# Pancreatic cancer in the MENA region, a bibliometric review

**DOI:** 10.3332/ecancer.2022.1380

**Published:** 2022-04-29

**Authors:** Hasan Nassereldine, Hussein Awada, Adel Hajj Ali, Mohammad Zeineddine, Zahy Abdul Sater, Yasser Shaib

**Affiliations:** 1 American University of Beirut Faculty of Medicine, Bliss Street, Hamra, Beirut, Lebanon; 2 American University of Beirut Global Health Institute, Bliss Street, Hamra, Beirut, Lebanon; 3 American University of Beirut Medical Center; Division of Gastroenterology, Bliss Street, Hamra, Beirut, Lebanon; * Authors corresponded equally and are considered first co-authors.; a https://orcid.org/0000-0003-2298-3098; b https://orcid.org/0000-0002-1445-7947; c https://orcid.org/0000-0002-7166-1071

**Keywords:** pancreatic cancer, MENA region, bibliometrics, research, oncology

## Abstract

**Background:**

Most Middle East and North Africa (MENA) countries record pancreatic cancer incidence rates that are above the world’s average. Reducing this burden requires evidence-based policies. This bibliometric review aims to examine the status of pancreatic cancer research in the MENA world, while systematically categorising publications across cancer care pathways.

**Methods:**

We searched Scopus, Medline and PubMed for peer-reviewed publications related to both pancreatic cancer and MENA countries by using controlled vocabulary and keywords. The results were screened for duplicates and later included in the analysis based on preset eligibility criteria. A structured data extraction form was used to collect data related to each article, its methodology, its cancer care pathway, funding status and authorship.

**Results:**

A total of 5,848 publications resulted from our search, from which 1,098 articles remained after applying the eligibility criteria. Trends show a steady increase in pancreatic cancer research by MENA. Case reports are the most common, whereas a lack in high-evidence clinical studies as well as public health and epidemiological research was evident. Most studies were not funded and had no female contributions. Funding, if present, came mostly from foreign states. There exists a much greater focus in research on diagnosis and treatment among other cancer care pathways. Most MENA-based studies did not involve collaborations with other countries. Country gross domestic product and population are both correlated to the research output.

**Conclusion:**

This bibliometric analysis identified significant gaps and limitations in pancreatic cancer research in MENA countries. Vital domains requiring research investment have also been highlighted as a first step towards evidence-based health policies.

## Introduction

Pancreatic cancer is one of the deadliest cancers worldwide and it is one of the leading causes of cancer-related death in developed countries [1–3]. The incidence of pancreatic cancer has been increasing over the years, with a reported incidence of 495,773 cases in 2020, making pancreatic cancer the 14th most diagnosed cancer worldwide [1]. The exact aetiologies of pancreatic cancer are still not well understood; however, there are well-known risk factors for the disease, such as smoking, obesity, diabetes mellitus, positive family history, alcohol use and dietary habits [4–7]. According to the 2020 GLOBOCAN report, pancreatic cancer was more reported in countries with high to a very high human development index (HDI) as compared to countries with a low HDI [1]. This is mainly because in countries with a high HDI, life longevity increases one’s risk of developing pancreatic cancer. Also, the availability and ease of access to medical care with an advanced and systematic cancer registration may explain the increased reported incidence in such countries [8, 9].

Despite not being one of the most common cancers, it has one of the highest mortality rates among cancers worldwide, with 466,003 people dying out of the 495,773 diagnosed, and only 6% of the patients surviving up to 5 years [1, 10]. The high mortality rates are attributed to the disease burden at the time of diagnosis [11]. Only 20% of the patients have a resectable mass on diagnosis [10, 11], and even with patients that undergo surgery, the 5-year survival is still very low (27%) [12].

The Middle East and North Africa (MENA) region consists of 21 countries (https://data.worldbank.org/country/ZQ). These countries display a wide variety in healthcare status, socio-economic stability, infrastructure development and resources [14, 15]. The incidence of pancreatic cancer in this area has increased over the years, with a slight male predominance [1], which sheds light on the importance of pancreatic cancer research in this area in order to tackle the increased morbidity and mortality from the disease. However, from a study conducted in 2016 concerning the average number of research publications per one million people in the MENA region, it has shown that the research output is only quarter of the world average [16]. This poses questions concerning the medical research on cancer, in general, and pancreatic cancers, specifically. These questions are very critical to the MENA world, since 12 of the 19 MENA countries record incidences of age-standardised risks (ASRs) that are greater than the world average in either males or females [17].

This study aims at approximating the number of research papers published from the MENA region concerning pancreatic cancer, presenting the most explored area of research in the topic and shedding light on the unexplored areas, hence raising the much-needed awareness regarding the medical research output on pancreatic cancer. We also aim at assessing the different epidemiological and demographic settings between these countries as we compare their research output and the trends in their productivity over the years.

## Methods

A comprehensive search of all pancreatic cancer publications between the year 2000 and 2020 was carried on PubMed, Medline and Scopus. The Boolean operators (AND, OR and NOT) in addition to [ad] were used for the search. The search terms used included ‘pancreatic cancer’, ‘pancreatic carcinoma’, ‘pancreatic oncology’, ‘pancreatic neoplasm’, ‘pancreatic adenocarcinoma’, ‘pancreatic metastasis’, ‘pancreatic malignancy’, ‘pancreatectomy’, ‘pancreatoduodenectomy’ and ‘Whipple’. Two authors then independently went over all the articles, and a third author was asked for further assessment, in case of disagreement between the first two authors, to select the appropriate match, remove any duplicates and identify the funding status; the presence or absence of a female author; the study design; the type of publication; the paper’s question; and the number of citations. If for a given author name the gender was not provided by Gender API, we manually confirmed the gender of the author through one of the following platforms: Google Scholar, ResearchGate, LinkedIn, Google images and academic institutional websites. As for the study design, it was determined based on what was provided in the methodology section of each paper. Additionally, the funding status was determined by checking the funding declaration section in each article.

The following 21 countries representing the MENA were included: Algeria, Bahrain, Djibouti, Egypt, Iraq, Iran, Jordan, Kuwait, Lebanon, Libya, Malta, Morocco, Oman, Palestine (West Bank and Gaza), Qatar, Saudi Arabia, Syria, Tunisia, United Arab Emirates, and Yemen.

For each of the above countries, we obtained the gross domestic product (GDP) and the population size from the World Bank [18]. In addition, the HDI was obtained in the selected countries [19]. In order to minimise the bias between Arab countries, we divided the number of publications of each country by its corresponding GDP and population size to obtain the number of publications per billion GDP and per million persons, respectively, similar to what was used before.

The analysis was conducted on both R and SPSS. Numerical data are reported as percentage and frequency. The data were analysed by chi-squared analysis and an alpha value less than 0.05 was considered significant. Graphical data are presented by a heat map, tables, pie charts, bar graphs and cluster graphs.

## Results

### Mapping the characteristics of the publications

**Figure 1.**
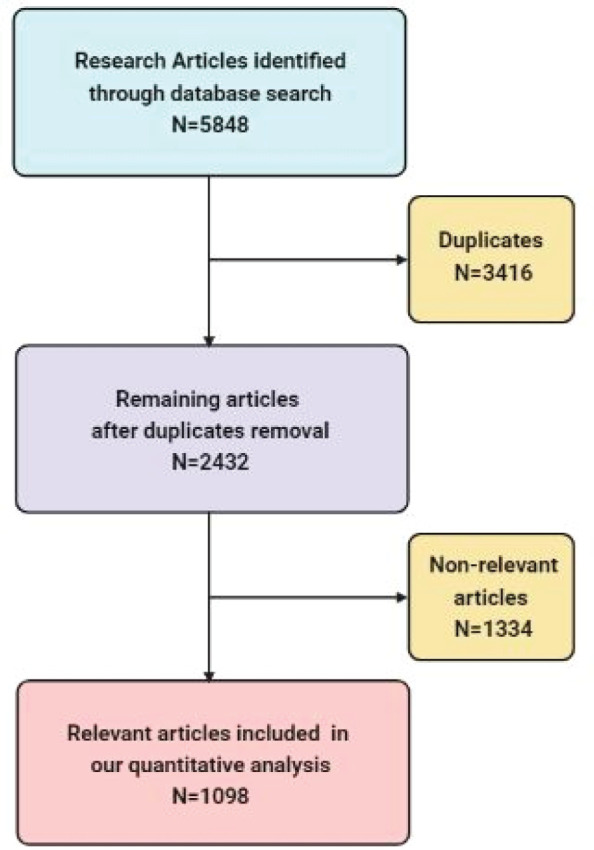
Flowchart of the articles’ selection process.

A total of 5,848 publications from our search were retrieved, of which 3,416 articles were excluded for being duplicates. The remaining 2,432 articles were screened by title and abstract and 1,098 were included in our study and 1,334 were excluded (Figure 1). Most of the included publications were in English (1,091), four in Turkish and three in French. We showed each country’s contribution in the MENA region to pancreatic cancer-related research.

**Figure 2.**
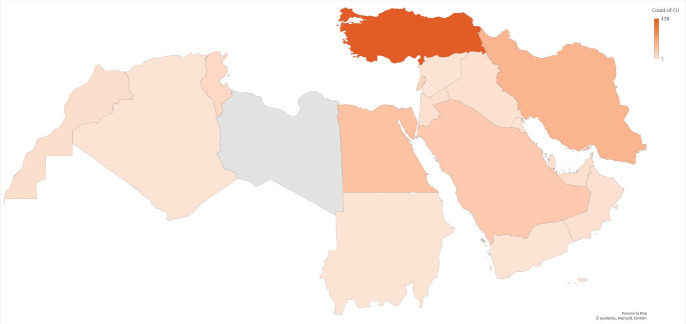
Contribution of each country in the MENA region to pancreatic cancer-related research.

**Figure 3.**
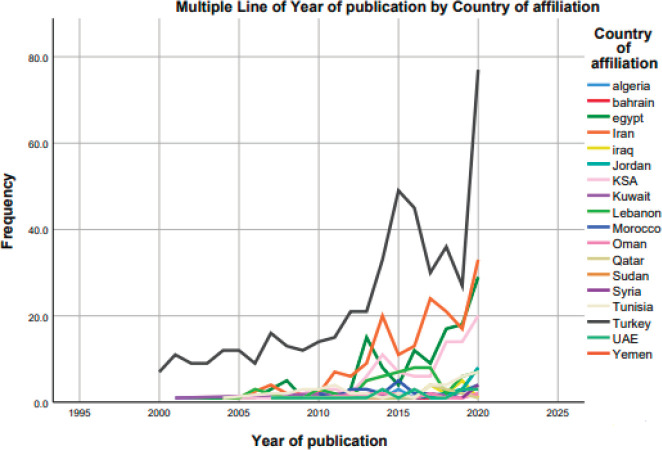
Trend of publications of each country in the MENA region across the years 2000–2020.

The highest contributing country is Turkey with the total number of publications reaching 478, followed by Iran (171), Egypt (129) and KSA (96) (Figure 2). Of all the MENA countries, the least contributing are Sudan and Yemen with each contributing 1 publication. Examining the temporal trends for pancreatic cancer research output in the MENA region, our data show that there is an increase in the number of outputs after 2005 across the region, with Turkey and Iran having the sharpest increases (Figure 3).

The articles we obtained were published in diverse journals with the highest journal publishing 24 articles, i.e., 2.2% of the total output of all researches of that journal. Table 1 presents the journals with 10 or more articles contributed by the MENA region on pancreatic cancer along with the journal’s current impact factor and quartile.

**Figure 4.**
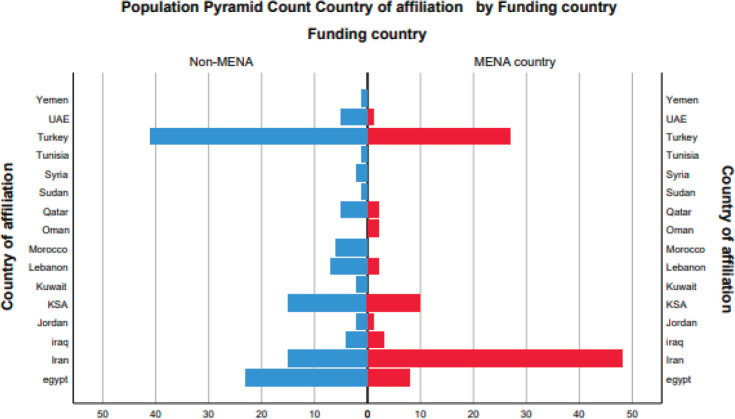
Difference in funding between each contributing MENA country.

**Figure 5.**
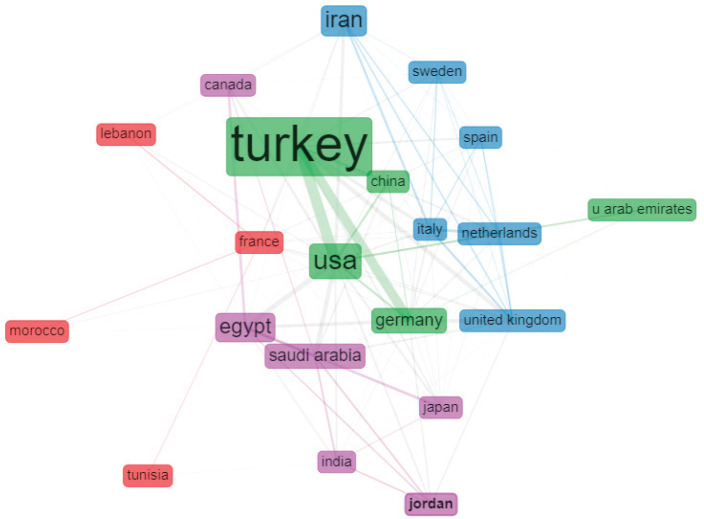
Graphical demonstration of the collaboration among the MENA countries and between the MENA countries and the world.

**Figure 6.**
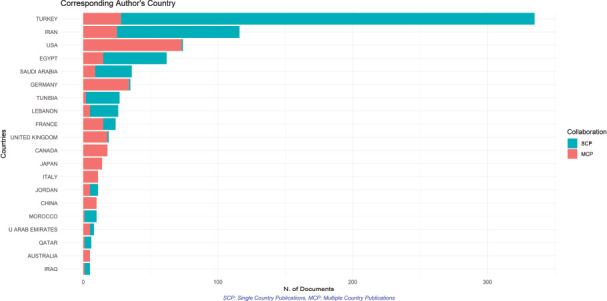
Bar chart showing the country of the corresponding author and its frequency.

Examining the differences in funding between each contributing MENA country (Figure 4), we found that 21.5% are funded, of which only 44.4% are funded by the MENA countries. Moreover, 12.2% of the total publications are funded by public sources and 9.2% by private sources. Multiple collaborations have taken place among the Arab countries and between the Arab countries and the world on the topic of prostate cancer (Figure 5); however, our analysis sheds light on a strong collaboration between Turkey and the USA and between Turkey and Germany, followed by Egypt and the USA. Corresponding authors originate mainly from Turkey, followed by Iran, USA and Egypt (Figure 6). In addition, our analysis shows that most of the MENA countries had more single country publications than multiple country publications.

### Types and topics of the studies

Case reports represented the highest percentage of the total publications (28.6%), with basic science research being the second reaching 20%. It is also the most funded among the rest of the study designs, representing 13.7% of the total funded publications. The rest of the study designs were as follows: 18.7% descriptive studies, 18.1% cohort studies, 3.7% cross-sectional studies, 3.6% case controls, 3.4% clinical trials, 3.4% quantitative studies and 0.1% qualitative studies. Moreover, 96.3% of the total number of publications is clinical and biomedical research as opposed to only 3.5% covering public health. Of all, 84% of the publications are research papers, which are the highest of all types of publications, with review papers ranking second at 11.4%.

**Figure 7.**
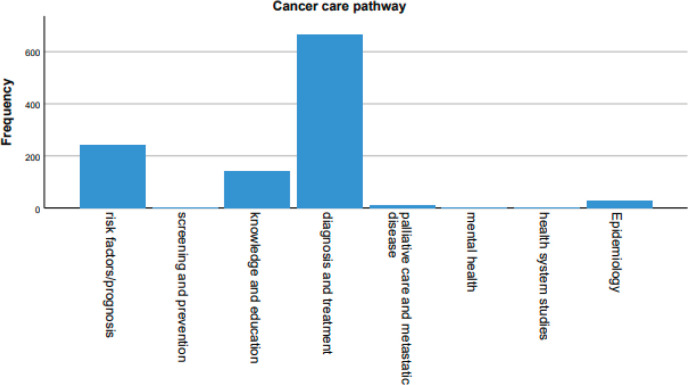
Various cancer care pathways addressed by the articles.

The different cancer care pathways addressed by the articles were explored (Figure 7). 60.5% of the articles cover diagnosis and treatment, followed by 22.2% covering risk factors and prognosis, 13% knowledge and education, 2.7% epidemiology, 0.8% palliative care and metastatic diseases, 0.3% screening and prevention, 0.2% health system studies and 0.1% mental health.

Our analysis of citations shows that only 5 articles had more than 100 citations, 26 articles between 50 and 100 and 326 publications with 0 citations.

### Publications and epidemiology

When adjusting the number of publications to the country’s GDP per capita, Turkey, Egypt and Iran have the greatest output. In addition, when analysing the number of publications with respect to population size, Lebanon, a small-sized population, ranks first, followed by Turkey. Moreover, there is a significant positive correlation between population size and research output and between GDP and research output, with a *p*-value < 0.001 each.

## Discussion

The search of the main biomedical bibliographic databases for peer-reviewed publications in the field of pancreatic cancer revealed only 1098 contributions by the MENA countries, which corresponds to approximately 1.2% of the total pancreatic cancer research published worldwide since the beginning of the 21st century. While we were expecting our search results to conform with the paucity in medical research output in our region of interest, we found that the gap with the rest of the world is much greater than the latter based on what is reported in the literature [16]. However, our review of the literature showed that similar studies focusing on other types of cancer research – like breast cancer – revealed a somewhat similar contribution (0.8%) by countries of this region, suggesting that this scarcity in research output in MENA countries pertains to the field of cancer in general [20].

On a positive note, our data show that the MENA countries have been experiencing a slow but steady growth in pancreatic cancer research output since the beginning of the current century. Furthermore, this growth has accelerated considerably over the last few years, especially when it comes to laboratory research work. Yet, when individual country contribution was put into perspective, we found that remarkable growth is in fact occurring in only four countries (Turkey, Iran, Egypt and KSA). Moreover, none of the MENA countries (the four countries included) had a sustained individual growth but rather more of an ‘unstable’ progress. This may be reflective of the volatility of the MENA region, in which political feuds, civil wars and regional instabilities hinder medical research by driving the brain drain to the west, while also shifting funding away from medical research towards more urgent domains [14, 16, 20, 21]. This finding was similarly explored in the scoping review by Abdul-Khalek *et al* [22], in which conflicted-affected MENA countries had the least contribution to breast cancer research.

The MENA countries still lag far behind the rest of the world, despite the overall yearly increase in total publications. Our results show that this trendy increase involves mainly basic research but not epidemiological or high-evidence clinical studies addressing any of the different cancer care pathways. Epidemiological studies are fundamental for increasing insight and addressing the relatively high rates of pancreatic cancer incidence of ASRs in some MENA countries [23]. High-evidence clinical research is also essential for setting unanimous and reliable guidelines to combat this disease, especially since most of the current clinical studies are being limited to low evidence case reports and other observational and descriptive research work [24]. Interestingly, our analysis showed that funding was a significant factor behind the discrepancy in the trend of research growth across the research fields. This, however, does not rule out that other factors, such as the lack of capacity, expertise and research collaboration, may also be implicated in this discrepancy. The deficit in these studies is worrisome because of the importance of epidemiological data as the base from which evidence-based public policies are inspired, and the role of high-quality clinical studies in ‘clinically’ guiding these policies [25, 26].

Public health is another aspect of pancreatic cancer research that needs massive improvement, as it represents only 3.5% of its total publications. While we assert the importance of biomedical research by calling for increasing its support and capacity, we also urge for backing public health research as it is as important in targeting the human population and health systems in an effort to reduce the burden of preventable morbidity and mortality [27].

The lack of funding for medical research remains a major hindering factor in the MENA region, as the number of MENA-based centres that fund cancer research represents only a slight minority compared with the rest of the globe [16, 28, 29]. Our findings showed that only 21.5% of the publications received financial support. It further showed that even within this minority of funded projects, the funding was more commonly (55.6%) received from non-MENA than MENA sources (except for Iran and Oman).

Our analysis showed that national GDP has a significant positive correlation with pancreatic cancer research output in MENA countries. This is expected, since the availability of funds allows its allocation towards medical research. But because of the ongoing regional conflicts, COVID-19-related financial constraints and long-standing economic instabilities affecting some MENA countries, efforts to allocate national funds towards medical and public health research may not be feasible even in some countries on the higher end of the GDP spectrum [14, 16, 30]. Alternatively, such limitations might be bypassed by establishing regional and international collaborations with other MENA and non-MENA institutions in order to increase the quantity and quality of their research output [31–33]. Mutual and commensal relationships can be set up, so that the involved parties complement each other’s deficits in funding, skills, expertise, patient population or other resources. Our investigation suggests that such relationships currently barely exist. Our findings demonstrated that the majority of MENA-based studies (based on the corresponding author’s affiliated country) were contributed by authors belonging to the same country. This may indicate that collaborations, if available, are initiated by non-MENA countries. In addition, 8 of the top 15 contributing countries for correspondence are of non-MENA origins. This further emphasises the lack of initiatives from MENA countries to perform research related to pancreatic cancer as well as to establish contacts with the rest of the world to bypass capacity limitations.

Finally, females remain underrepresented in medical research in the MENA world, despite the worldwide trend towards increasing their involvement [34]. Cultural factors persist up to this day, with our findings showing that just 60% of pancreatic cancer publications included at least 1 female author. Females make up around 70% of the global healthcare workers, and increasing their contribution to areas requiring workforce expansion can help accelerate the growth in pancreatic cancer research across the MENA region [35].

This study, however, has a few limitations. Due to some of the ongoing conflicts in some countries in the MENA region, the study results may not reflect the actual willingness of some countries to conduct research. Additionally, unlike systematic reviews, bibliometric reviews emphasise the methodology and research design of the article instead of the results of the research. Moreover, one common limitation with bibliometric reviews is that researchers are reliant on the indexing of the databases used. One last limitation is that the degree of agreement was not measured between the appointed authors responsible for the review.

## Conclusion

With pancreatic cancer being among the most aggressive cancers worldwide, and with its relatively high incidence in some MENA countries, improving its regional research activity becomes a necessity in order to reduce unfortunate morbidity and mortality. While there is a general increase in the pancreatic cancer research output in the MENA world, we have noted significant quantitative and qualitative limitations and gaps that require improvements in order to guide context-specific regional health policies.

## Funding

The authors did not receive any funding.

## Conflicts of interest

The authors declare no conflicts of interest.

## Figures and Tables

**Table 1.** Journals with 10 or more publications concerning pancreatic cancer in the MENA countries.

	Frequency	Percent	Quartile	IF
Asian Pacific Journal of Cancer Prevention	23	2.1	N/A	N/A
Hepato-Gastroenterology	16	1.5	N/A	N/A
International Journal of Surgery Case Reports	21	1.9	N/A	N/A
Journal of the Balkan Union of Oncology	14	1.3	Q4	1.695
Journal of Gastrointestinal Cancer	19	1.7	N/A	N/A
Journal of Oncology Practice	20	1.8	Q2	3.551
Pancreas	14	2.3	Q3	2.920
Pancreatology	24	2.2	Q2	3.629
PLoS One	11	1.0	Q2	2.740
Scientific Reports	10	0.9	Q1	3.998
Turkish Journal of Gastroenterology	22	2.0	Q4	1.111
World Journal of Gastroenterology	10	0.9	Q2	3.665
